# Experimental Investigation of Three-Dimensional Multi-Directional Piezoelectric Wind Energy Harvester

**DOI:** 10.3390/s24237757

**Published:** 2024-12-04

**Authors:** Zonghao Chen, Xiaohan Liao, Shen Li, Shu Pu, Pengfei Li, Dingkun He, Yizhou Ye, Xuefeng He

**Affiliations:** 1 College of Optoelectronic Engineering, Chongqing University, Chongqing 400044, China; chenzonghao98@163.com (Z.C.);; 2 Chengdu CAIC Electronics Co., Ltd., Chengdu 610000, China; 3 Chongqing Construction Science Research Institute Co., Ltd., Chongqing 400010, China

**Keywords:** energy harvesting, wind-induced vibration, three-dimension, multi-direction, piezoelectricity

## Abstract

The wind-induced vibration energy harvester is a type of ideal power source for wireless sensor nodes. To adapt to the uncertainty of wind direction in natural environments, this paper proposes a three-dimensional multi-directional piezoelectric wind energy harvester (WEH), whose bluff body is an external shell with the shape like a lampshade, supported by three internal piezoelectric composite beams. A harvester prototype was made using 3D printing technology, and its multi-directional energy harvesting characteristics were systematically tested in a wind tunnel. Experiments show that it can harvest wind energy from any direction in three-dimensional space. When the wind speed is about 15 m/s and the wind direction changes in the horizontal plane, the minimum to maximum total average output power ratio is about 0.84. This work provides an experimental basis for the future development of three-dimensional multi-directional WEHs to some extent.

## 1. Introduction

With the advent of the “Internet of Everything” era, wireless sensor network technology attracts increasing attention in recent years. Wireless sensor nodes are the fundamental building blocks for constructing wireless sensor networks. The current power sources for these nodes are predominantly traditional chemical batteries. These batteries have disadvantages such as the limited lifespan, high maintenance costs, and environmental pollution, hindering the wide applications of wireless sensor networks in fields such as environmental monitoring. Wind energy is a readily accessible and clean energy source in the natural environment, and small-scale wind energy harvesters (WEHs), which convert the ambient wind energy into electrical energy, have advantages such as a long lifespan, being maintenance-free, and producing zero pollution. WEHs can function as either replacements or supplementary power sources for wireless sensor nodes, significantly extending the lifespan of power supplies while reducing environmental pollution [1,2]. Small WEHs mainly include wind turbines and wind-induced vibration (WIV) energy harvesters. The blades of the wind turbines rotate due to the action of the wind, converting rotational energy into electrical energy through electromagnetic induction. WIV WEHs transform wind energy into vibrational energy utilizing the wind-induced vibration phenomena and then convert vibrational energy into electrical energy through electromechanical transducers. Unlike wind turbines, WIV WEHs do not have rotors, offering advantages of compact structure and easy miniaturization [3,4]. WIV WEHs can be classified into several types according to their electromechanical conversion mechanisms, including piezoelectric, electromagnetic, electrostatic, and triboelectric harvesters [5]. Notably, piezoelectric wind energy harvesters (PWEHs) based on WIVs have advantages such as the structural simplicity and high energy conversion efficiency, and therefore have received the most attention [6,7]. WIV PWEHs based on vortex-induced vibration (VIV), galloping, and flutter have been developed [8,9,10].

Most WIV WEHs are designed to capture wind energy from a specific direction. Their output powers decrease sharply when the wind direction deviates from this direction. In natural environments, wind direction frequently changes, preventing uni-directional WIV WEHs from providing stable power to electrical loads. Therefore, it is of great importance to develop multi-directional WIV WEHs. Zhao et al. [11] replaced the traditional straight cantilever beam with a curved elastic beam, using the wind load on the curved structure and the beam’s elastic properties to enable the wind energy to be harvested from multiple directions. They tested the wind energy harvesting performance in four directions. Tang et al. [12] proposed a multi-directional PWEH, mainly consisting of a wind vane, a piezoelectric beam, an S-shaped turbine, a striking rod, and fixed supports. Under the action of wind from various directions, both the S-shaped turbine and the striking rod strike the piezoelectric beam, generating electrical output. However, this harvester has a relatively complex structure and is relatively difficult to miniaturize. Shi et al. [13] proposed a multi-directional PWEH based on vortex-induced vibration (VIV), which achieves omnidirectional wind energy harvesting within the plane by employing the crossed-coupling of dual piezoelectric composite beams. Li et al. [14] designed a VIV PWEH capable of capturing omnidirectional wind energy within the plane. They developed a full theoretical model, and both theoretical and experimental results confirm that the harvester can effectively capture omnidirectional wind energy within the plane. Li et al. [15] also proposed the first planar omnidirectional PWEH based on flutter. Xin et al. [16] replaced the traditional thin rectangular cantilever beam with a cylindrical beam, enabling omnidirectional wind energy harvesting within a plane. However, it should be noted that none of the studies mentioned above conducted systematic experimental research on wind energy harvesting characteristics when the wind direction varies in a three-dimensional space. Therefore, for application scenarios where the wind direction changes in a three-dimensional space, such as for wireless tilt sensor nodes deployed on steel truss transmission towers, how to improve the wind direction adaptability of WIV WEHs is still an urgent issue that needs to be solved.

This paper proposes a WIV PWEH capable of collecting wind energy from any direction in a three-dimensional space with an external bluff body shell shaped like a lampshade and supported by three piezoelectric composite beams. The equations of motion and coupled circuit equations are presented and then used to analyze the main parameters that determine the electrical outputs. Components were fabricated using 3D printing technology and assembled into a harvester prototype. Finally, the output performance of the prototype was systematically tested when the wind direction changed in a three-dimensional space in a wind tunnel. The experiments verify that the proposed PWEH can convert wind energy from any direction in three-dimensional space into electrical energy.

## 2. Harvester Structure and Simulation Analysis

### 2.1. Harvester Structure

**Figure 1:**
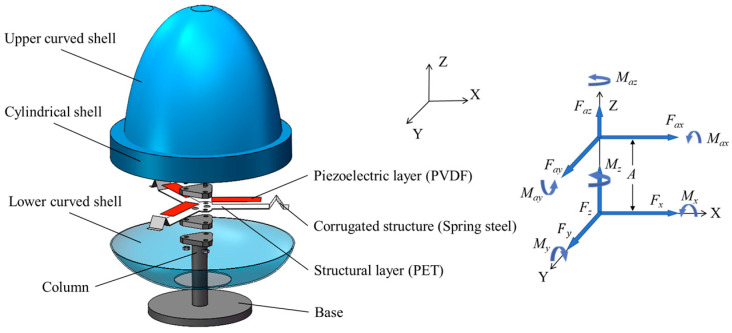
Schematic of the three-dimensional multi-directional wind energy harvester.

As shown in Figure 1, the bluff body of the proposed three-dimensional multidirectional WIV PWEH is a hollow rigid shell similar to a lampshade. The bluff body is fixed to a column by three piezoelectric composite beams which are evenly distributed along the circumferential direction. The bluff body consists of an upper curved shell, a connecting plate, a cylindrical shell, and a lower curved shell. The connecting plate is used to fix the upper curved shell and cylindrical shell together, while the lower curved shell is fixed to the bottom of the cylindrical shell. The diameter of the upper curved shell is smaller than that of the cylindrical shell. One end of each piezoelectric composite beam is fixed to the column, while the other end is fixed to the inner wall of the bluff body’s cylindrical shell through a corrugated structure. By increasing the length of the piezoelectric composite beam and the diameter of the lower cylindrical shell, the cut-in wind speed of the harvester can be reduced. There is a hole in the center of the lower curved shell and one end of the column is in the bluff body shell. The other end of the column extends out of the bluff body shell and fixed to the base through the hole. The bluff body shell of the harvester offers protection for the piezoelectric composite beams, and the central hole in the lower curved shell can be sealed with a flexible rubber membrane.

### 2.2. Working Principle

When the wind blows from any direction in three-dimensional space and the wind speed is higher than the cut-in speed, the bluff body experiences WIV, causing the piezoelectric composite beams to vibrate. The piezoelectric layers of these beams produce alternating potential differences which can be used to power the electrical loads. The wind loads on the bluff body can be expressed as the aerodynamic loads acting at the aerodynamic center, including the aerodynamic forces $F_{ax}$, $F_{ay}$, and $F_{az}$ and the aerodynamic moments $\;M_{ax}$, $M_{ay}$, and $M_{az}$. Since the bluff body is axisymmetric, the aerodynamic center is located on the *z* axis. The three piezoelectric composite beams, serving as the support structure, are evenly arranged along the circumference, with their neutral axes intersecting at a point on the *z* axis, which is the stiffness center of the harvester. Since the upper curved shell of the bluff body is longer and the lower curved shell is shorter, although the aerodynamic center and the stiffness center are both on the *z* axis, the aerodynamic center is located at a higher position than the stiffness center. Assuming the distance between the aerodynamic center and the stiffness center is *A*, the aerodynamic loads applied at the aerodynamic center of the bluff body shell can be equivalently shifted to the stiffness center. The equivalent aerodynamic loads at the stiffness center can be expressed as

(1) $$F_{x}=F_{ax},\;F_{y}=F_{ay},\;F_{z}=F_{az},\;M_{x}=M_{ax}+AF_{ay},\;M_{y}=M_{ay}-AF_{ax},\;M_{z}=M_{az}$$

The aerodynamic loads at the aerodynamic center can be determined through computational fluid dynamic (CFD) simulations or wind tunnel tests. The equations of motion and coupled circuit equations for the harvester are given by

(2) $$\left\{\begin{array}{c} \left[M\right]\left\{\ddot{u}\right\}+\left[C\right]\left\{\dot{u}\right\}+\left[K\right]\left\{u\right\}+\left[\Theta \right]\left\{V\right\}=\left\{F\right\}\\ \left[C_{p}\right]\left\{\dot{V}\right\}+\left[R\right]\left\{V\right\}-\left[\Theta \right]\left\{\dot{u}\right\}=0 \end{array}\;\right.$$

where $\left[M\right]$, $\left[C\right]$, $\left[K\right]$, $\left[C_{p}\right]$, $\left[\Theta \right]$, and $\left[R\right]$ represent the inertia matrix, mechanical damping matrix, stiffness matrix, capacitance matrix, electromechanical coupling matrix, and impedance matrix. The displacement vector is $\left\{u\right\}={\left[u_{x}\;u_{y}\;u_{z}\;\vartheta_{x}\;\vartheta_{y}\;\vartheta_{z}\right]}^{T}$, where $u_{x}$, $u_{y}$, and $u_{z}$ represent the displacements along the *x*, *y*, and z axes, and $\vartheta_{x}$, $\vartheta_{y}$, and $\vartheta_{z}$ represent the rotational angles of the center of mass around the *x*, *y*, and *z* axes, respectively. The vector $\left\{V\right\}={\left[V_{1}\;V_{2}\;V_{3}\right]}^{T}$ denotes the voltages of the piezoelectric layers, where $V_{i}\left(i=1,2,3\right)$ is the output voltage of the piezoelectric layer on the *i*th piezoelectric composite beam. The aerodynamic force vector is $\left\{F\right\}={\left[F_{x}\;F_{y}\;F_{z}\;M_{x}\;M_{y}\;M_{z}\right]}^{T}$.

When the wind speed and the wind direction remain unchanged, the harvester experiences stable WIV and the aerodynamic forces and moments can be represented as the superpositions of static and dynamic loads. In this case, the harvester’s motion is the superimposition of static and dynamic motions. For a PWEH, there is no electrical output corresponding to static motion. Therefore, only the electrical output caused by dynamic motion should be considered. The PWEH shown in Figure 1 has a high translational stiffness in the horizontal plane. During WIV, $u_{x}$ and $u_{y}$ are small, and $\vartheta_{z}$ contributes little to the electrical outputs. Therefore, the electrical outputs of the three piezoelectric composite beams are mainly determined by $u_{z}$, $\vartheta_{x}$, and $\vartheta_{y}$, which are in turn primarily affected by $F_{z}$, $M_{x}$, and $M_{y}$. According to Equation (1), for the same bluff body shell, as *A* increases, the relative effects of $F_{ay}$ on $M_{x}$ and $F_{ax}$ on $M_{y}$ become more pronounced. Thus, increasing *A* can enlarge the amplitudes of $\vartheta_{x}$ and $\vartheta_{y}$, subsequently increasing the electrical outputs. This accounts for the different lengths of the upper and lower curved shells in the harvester, as shown in Figure 1. In summary, $F_{az}$, $M_{ax}$, and $M_{ay}$ have significant effects on electrical output, $M_{az}$ has a small, negligible effect on electrical output, and the effects of $F_{ax}$ and $F_{ay}$ on electrical output increases with *A*. In addition, by placing the piezoelectric composite beams inside the bluff body shell, the influence of external environmental factors such as sunlight, rain, and sand on the durability of the piezoelectric composite beam can be effectively reduced.

The bluff body shell is fixed to the column by three piezoelectric composite beams uniformly arranged along the circumferential direction through corrugated structures. The corrugated structures make it difficult to obtain the analytical expression of the stiffness matrix $\left[K\right]$. Finite element analysis is used to analyze the dynamic characteristics of the harvester in the following, with the geometrical and material parameters listed in Table 1.

First, the three-dimensional model of the harvester was established using SolidWorks 2011, and then the geometric model was imported into the finite element analysis software Ansys 12.0. Mesh generation was performed for the piezoelectric composite beam and the bluff body. Since deformation and stress are larger at the ends and connection points of the beams, a finer mesh was used in these regions. The bluff body, being larger in size and experiencing minimal deformation, was meshed using a coarser mesh. Local refinement mesh was adopted at the connection region near the piezoelectric composite beam. An unstructured mesh was employed throughout to reduce computation time while maintaining accuracy. The free ends of the piezoelectric composite beams were rigidly connected to the inner side of the bluff body, and all degrees of freedom of the supporting column were set as zero. Finally, a modal analysis was conducted to calculate the natural frequencies and corresponding vibration modes of the harvester.

**Figure 2:**
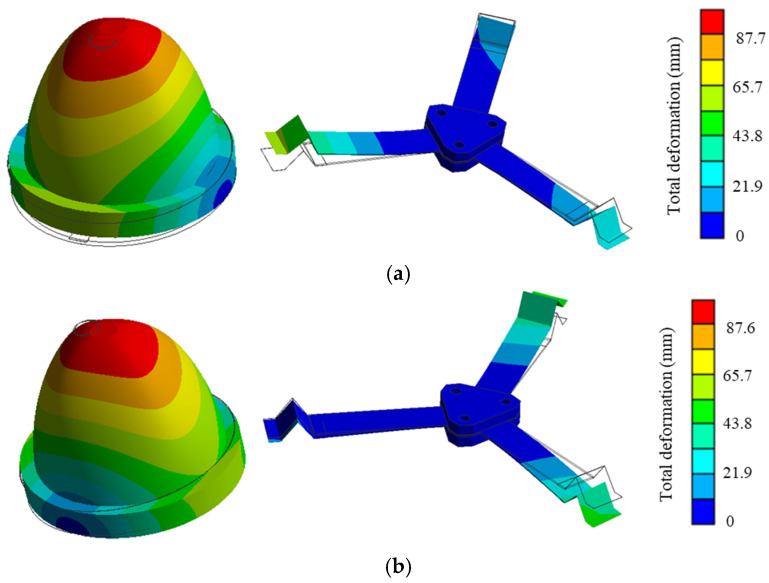
The first two mode shapes: (**a**) first mode (12.09 Hz); (**b**) second mode (12.44 Hz).

The simulated first two vibration modes of the harvester are shown in Figure 2. The first and the second modes involve rotation around two mutually perpendicular axes in the horizontal plane. The simulated first two natural frequencies are 12.09 Hz and 12.44 Hz, respectively, which are very close, indicating that the harvester’s dynamic characteristics exhibit good axial symmetry.

**Figure 3:**
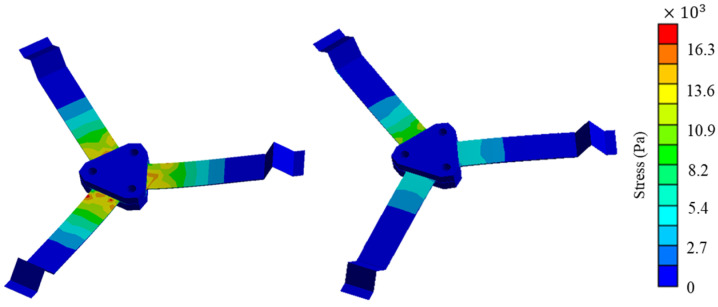
Stress distribution of the first two vibration modes.

Figure 3 shows the stress distribution on the surface of the piezoelectric composite beam corresponding to the two vibration modes. The stress is larger near the root of the composite beam, exhibiting a clear stress concentration, and as the distance from the root increases, the stress on the beam surface gradually decreases.

## 3. Experimental Results Analysis

### 3.1. Prototype Fabrication and Experimental Setup

**Figure 4:**
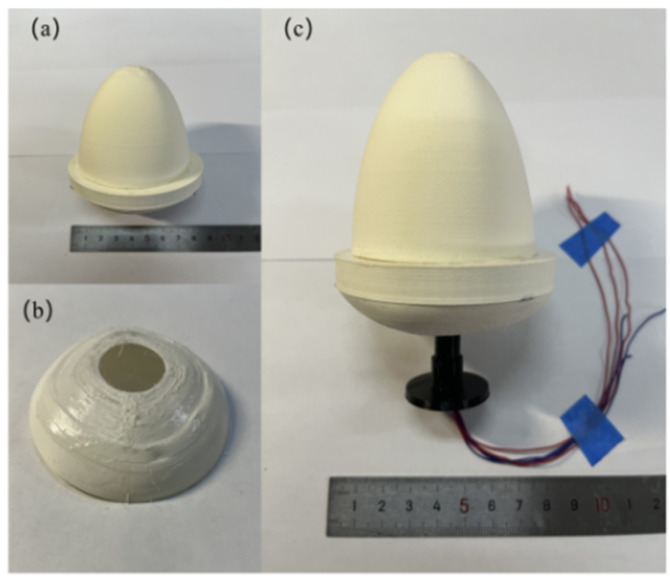
Photographs of the harvester: (**a**) upper curved shell; (**b**) lower curved shell; (**c**) assembled prototype.

The bluff body shell, column, and base were fabricated using 3D printing technology. To reduce mass and improve output power, the wall thickness of the bluff body shell is set to 0.4 mm. As listed in Table 1, the bottom diameter and height of the upper curved shell are 60 mm and 50 mm, respectively. The diameter and height of the cylindrical shell are 70 mm and 10 mm, respectively. To protect the piezoelectric composite beams, a lower curved bottom plate is positioned at the bottom of the cylindrical shell, with a diameter of 70 mm and a height of 15 mm. A straight segment of the piezoelectric composite beam is made of PET (polyethylene terephthalate) material and a single layer of PVDF (polyvinylidene fluoride) piezoelectric film. The PVDF piezoelectric film is attached to the upper surface of the PET beam, and the effective length of the piezoelectric composite beam is 20 mm. The corrugated structure at the end of the piezoelectric composite beam is triangular, made from 0.05 mm thick 65 Mn spring steel. The length, width, and height of the corrugated structure are 6, 6 and 4 mm, respectively. Figure 4 shows the photos of the prototype.

**Figure 5:**
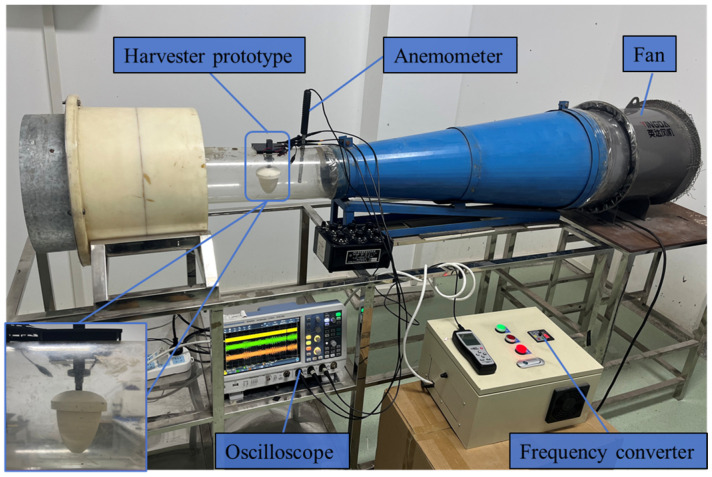
Wind tunnel experimental platform.

The performance of the prototype was tested in a wind tunnel, as shown in Figure 5. The wind speed is controlled by a frequency converter and monitored by a thermosensitive anemometer. The output voltages of the three piezoelectric composite beams are measured by an oscilloscope through three probes with the impedance of 10 MΩ, respectively. The oscilloscope displays the waveforms and the root mean square (RMS) values of the output voltages of the three piezoelectric composite beams in real time.

**Figure 6:**
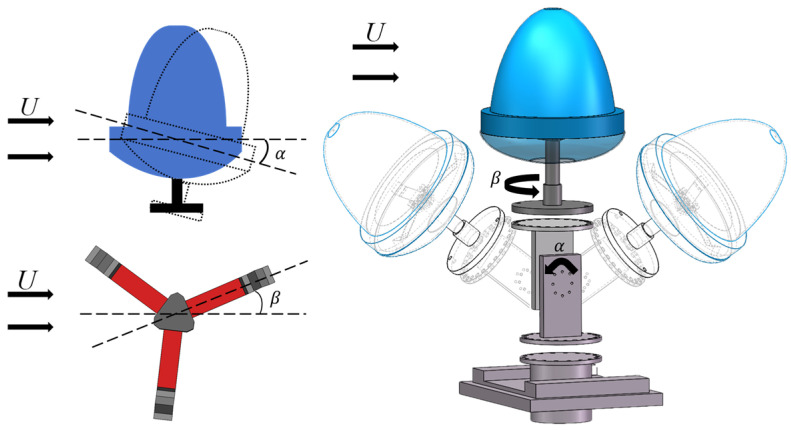
Angle adjuster.

To characterize the output performance of the harvester for different wind directions in three-dimensional space, the angle between the wind direction and the horizontal plane is defined as α, and the angle between the projection of the wind direction on the horizontal plane and the *x* axis is β. By varying α and β, the performance of the harvester for any wind direction in three-dimensional space can be measured. An angle adjuster was used to precisely adjust the relative direction of the wind with respect to the harvester. Figure 6 shows a schematic of the angle adjuster, which consists of two main parts: the α angle adjustment section and the β angle adjustment section. The α angle is adjusted by fixing two rectangular plates at different angular positions using screws. The β angle is adjusted by changing the hole positions on two circular plates.

### 3.2. Experimental Results

First, the experimental results showed that, when the wind speed is 6.5 m/s, the equivalent impedance of each piezoelectric composite beam is about 46 MΩ. To evaluate the output performance of the harvester for different wind directions, three electrical loads with the resistance of 46 MΩ are series connected with the three piezoelectric composite beams, respectively. During the experiments, the angle adjuster was used to set α to ±90°, ±60°, ±45°, ±30°, and 0°, while for each α, β was set to 0°, 20°, 40°, and 60°, respectively. The wind speed was adjusted for each specific combination of α and β to measure the corresponding electrical output. According to the structural symmetry, the electrical outputs with β increasing from 0° to 360° can be obtained by those measured with β increasing from 0° to 60°. If the experimental RMS voltage across the resistor connected to a beam is *V_rms_*, the average output power of this beam is obtained by $P_{ave}=V_{rms}^{2}/R$, where R=46 MΩ. The total average output power of the harvester is obtained by adding the average output power of the three beams.

**Figure 7:**
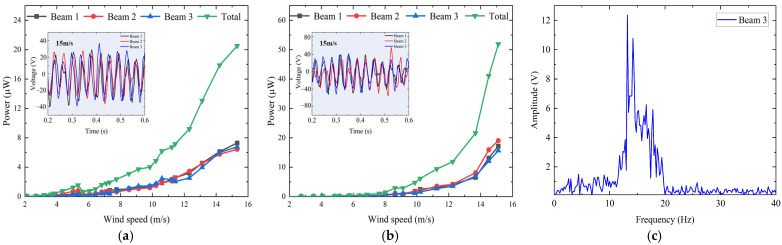
Output performance with α = ±90°: (**a**) average output power versus wind speed with α=90°; (**b**) average output power versus wind speed with α=−90°; (**c**) FFT of Beam 3’s output voltage with α=90° at 15 m/s.

When α=−90° (with the wind direction from bottom to top) and α=90°, the wind direction corresponding to different β values is the same. When the wind speed increased from about 2 m/s to approximately 15 m/s, the experimental average output powers of three beams and the total average output power of the harvester are shown in Figure 7a,b. For α=−90°, the cut-in wind speed is around 7 m/s, and the average output powers of three beams and the total average output power of the harvester increase with the wind speed, as shown in Figure 7a. When the wind speed reaches about 15 m/s, the total average output power reaches its maximum value of 51.9 μW. For α=90°, the cut-in wind speed is about 4 m/s, and when the wind speed increases to around 15 m/s, the total average output power reaches its maximum value of 20.5 μW, as shown in Figure 7b. The voltage waveforms of the three beams voltage at 15 m/s are also given in Figure 7a,b. Figure 7c gives the Fourier transform (FFT) of Beam 3’s output voltage and the frequency corresponding to the maximum output voltage is about 13.6 Hz.

**Figure 8:**
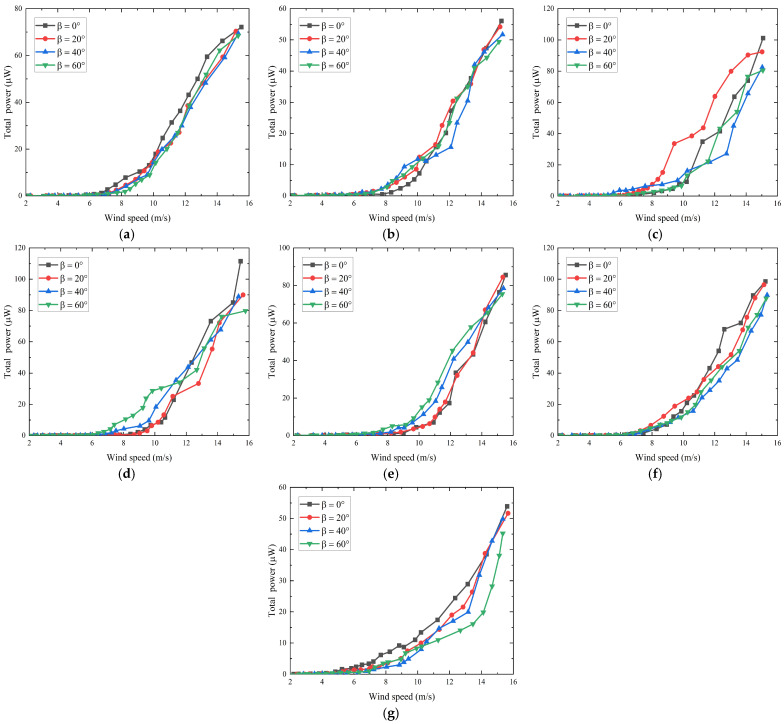
Total average output power versus wind speed for different directions: (**a**) α=−60°; (**b**) α=60°; (**c**) α=−45°; (**d**) α=45°; (**e**) α=−30°; (**f**) α=30°; (**g**) α=0°.

The experimental relationships between total average output power and wind speed for different wind directions is shown in Figure 8. For α=−60°, the cut-in wind speed for different β is approximately 7.5 m/s, with the total average output power for the optimized load of 72.1, 70.3, 69.4, and 68.4 μW, respectively. For α=60°, the cut-in wind speed for different β is about 8 m/s, with the total average output power of 56.0, 54.2, 51.7, and 49.4 μW, respectively. For α=−45°, the cut-in wind speed for different β is approximately 6.5 m/s, with the total average output power of 101.2, 90.3, 82.5, and 80.5 μW, respectively. For α=45°, the cut-in wind speed for different β is about 8 m/s, with the total average output power of 111.5, 90.0, 88.9, and 79.7 μW, respectively. For α=−30°, the cut-in wind speed for different β is approximately 8.5 m/s, with the total average output power of 85.6, 84.4, 78.5, and 75.4 μW, respectively. For α=30°, the cut-in wind speed for different β is about 7.7 m/s, with the total average output power of 98.6, 96.5, 89.8, and 87.7 μW, respectively. For α=0° (with the wind direction perpendicular to the side wall of the bluff body shell), the cut-in wind speed for different β is approximately 7 m/s, with the total average output power of 53.9, 51.7, 49.9, and 45.2 μW, respectively.

**Figure 9:**
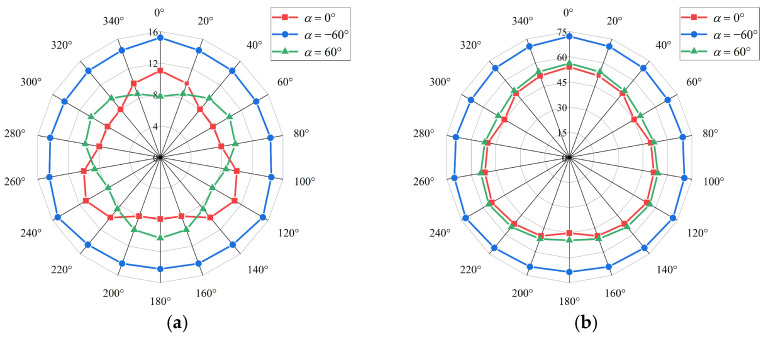
Effect of wind direction on total average output power: (**a**) wind speed of 10 m/s; (**b**) wind speed of 15 m/s.

Figure 9 shows the total average output power for different wind directions when the wind speed is approximately 10 and 15 m/s for α=−60°, 0°, and 60°. At a wind speed of 10 m/s, when α is −60°, 0°, and 60°, the minimum to maximum total average output power ratio is about 0.93, 0.78, and 0.75, respectively. At a wind speed of 15 m/s, when α is −60°, 0°, and 60°, the minimum to maximum total average output power ratio is 0.88. 0.84, and 0.91, respectively.

**Figure 10:**
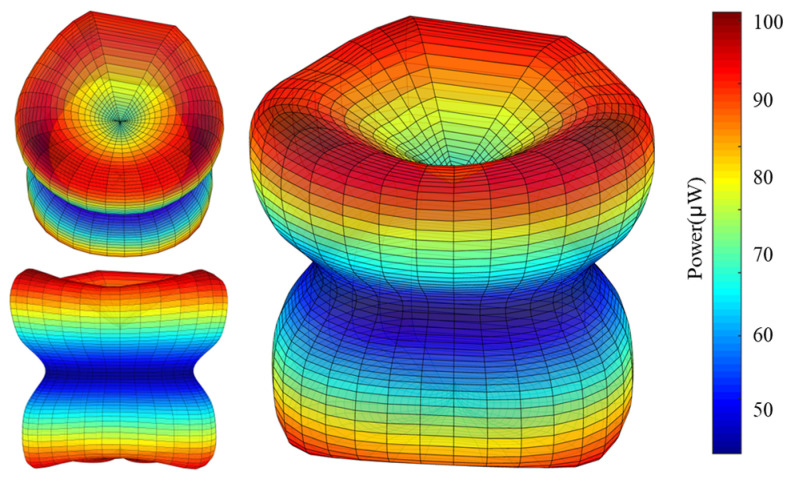
Experimental total average output power in different directions in a 3D space.

To visually observe the dependence of total average output power on wind direction in three-dimensional space, for any wind direction defined by α and β, by using r to represent the corresponding total average output power, a point with the spherical coordinate of $\left(r,\alpha ,\beta \right)$ can be used to describe the relationship between the total average output power and wind direction. The dependance of total average output power on wind directions in three-dimensional space obtained from the previous experimental results is shown in Figure 10. For a specific α, the total average output power does not vary much with different β. The total average output power is relatively higher for the wind directions with α = ±45°, and relatively lower for the wind directions with α = ±90°. When the absolute value of α increases from 0° to 90°, the total average output power first increases and then decreases.

According to the above experiments, when the wind direction changes arbitrarily in a three-dimensional space, the harvester can effectively capture wind energy, and the total average output power generally increases with increasing wind speed. When the angle α between the wind direction and the horizontal plane remains fixed, the change in β within the plane has relatively small influence on the total average output power. The total average output power is relatively high when α = ±45° and relatively low when α = ±90°.

## 4. Conclusions

This paper proposes a three-dimensional multi-directional PWEH to improve directional adaptability. The shape of its bluff body shell is like a lampshade, and the electromechanical conversion unit consists of three piezoelectric composite beams. The working principle of the harvester is analyzed, and the three-dimensional multi-directional wind energy harvesting performance of the prototype is tested in a wind tunnel. The experimental results indicate that the total average output power of the harvester increases with wind speed, and the harvester is capable of collecting wind energy from any direction in a three-dimensional space. The total average output power is relatively high when the angle between the wind direction and the horizontal plane is 45° and relatively low when the angle is 90°. At a wind speed of 15 m/s, the minimum to maximum total average output power ratio is approximately 0.84 when the wind direction changes in the horizontal plane.

The results in this work provide experimental evidence for the future development of three-dimensional multi-directional WEHs. The shape, size, and material of the bluff body, the shape and material of the elastic beam, the material of the piezoelectric layer, and the distance between the aerodynamic center and the stiffness center have significant effects on the cut-in wind speed, wind direction dependence, and output power. To optimize these parameters, the relationships between the aerodynamic loads and wind speed/direction should be extracted through wind tunnel experiments or computational fluid dynamics simulations in the future.

## Figures and Tables

**Table 1:** Geometrical and material parameters in simulations.

Parameter	Value	Materials	Young’s Modulus	Density	Poisson’s Ratio
Diameter of upper curved shell (mm)	60	Light weight PLA	1.94 GPa	500 kg/m^3^	0.40
Height of upper curved shell (mm)	50				
Diameter of lower curved shell (mm)	70				
Height of lower curved shell (mm)	15				
Diameter of cylindrical shell (mm)	70				
Height of cylindrical shell (mm)	10				
Length of corrugated spring beam (mm)	6	Spring steel	178 GPa	7850 kg/m^3^	0.28
Height of corrugated spring beam (mm)	4				
Thickness of corrugated spring beam (μm)	50				
Length of piezoelectric layer (mm)	20	PVDF film	2.6 GPa	1780 kg/m^3^	0.38
Thickness of piezoelectric layer (μm)	100				
Width of piezoelectric layer (mm)	6				
Thickness of PET layer (μm)	300	PET film	4 GPa	1400 kg/m^3^	0.38
Length of PET layer (mm)	30				
Thickness of supporting bracket (mm)	2	PLA	1.94 GPa	1240 kg/m^3^	0.40
Piezoelectric strain constant of PVDF (PC/N)	−33	**/**	**/**	**/**	**/**
Relative permittivity (*ε*/*ε*_0_)	10.5				
Bluff body mass (g)	3.32				

## Data Availability

Data are contained within the article.
